# Tumor-associated macrophages in colon cancer immunotherapy: mechanisms, natural product interventions, and microenvironment remodeling

**DOI:** 10.3389/fimmu.2025.1642091

**Published:** 2025-08-12

**Authors:** Qingman He, Li Xiang, Yuanyuan Luo, Rongrong Wang, Chuan Zheng, Yongxiang Gao, Huan Yao

**Affiliations:** ^1^ Department of Rheumatology and Immunology, Hospital of Chengdu University of Traditional Chinese Medicine, Chengdu, China; ^2^ College of Basic Medicine, Chengdu University of Traditional Chinese Medicine, Chengdu, Sichuan, China; ^3^ Sichuan Provincial Engineering Technology Research Center of Natural Small Molecule Drug, Tianfu Traditional Chinese Medicine (TCM) Innovation Harbor, Chengdu University of Traditional Chinese Medicine, Chengdu, China; ^4^ Traditional Chinese Medicine (TCM) Regulating Metabolic Diseases Key Laboratory of Sichuan Province, Hospital of Chengdu University of Traditional Chinese Medicine, Chengdu, China; ^5^ Sichuan Provincial Engineering Research Center of Innovative Re-development of Famous Classical Formulas, Tianfu Traditional Chinese Medicine (TCM) Innovation Harbour, Chengdu University of Traditional Chinese Medicine, Chengdu, China

**Keywords:** tumor-associated macrophages, colon cancer, phytochemicals, immune checkpoint, nanoparticle delivery

## Abstract

Colon cancer persists as a major global health burden due to therapy resistance and metastasis, with tumor-associated macrophages (TAMs) in the microenvironment driving progression through immune evasion and angiogenesis. This review highlights plant-derived therapeutics targeting TAMs to disrupt protumor signaling. Key phytochemicals (e.g., Curcumin, Cucurbitacin B, Astragaloside IV) suppress M2 polarization via NF-κB/STAT3 inhibition, block VEGF/HIF-1α-mediated angiogenesis, and enhance antitumor immunity by downregulating PD-L1. Cannabidiol, Hydroxygenkwanin regulate TAM metabolism. Dietary agents like sulforaphane and β-glucans modulate TAM-gut microbiome crosstalk. Nanoparticle-encapsulated phytochemicals enhance TAM-targeted delivery, while clinical translation requires standardized phytopreparations and biomarker-guided trials. We propose integrating validated botanical adjuvants (e.g., Fucoidan for TLR4 inhibition, dihydroisotanshinone I for CCL2 suppression) with immunotherapies to remodel immunosuppressive niches. Phytotherapy offers a multifaceted strategy to overcome TAM-driven therapeutic barriers in colon cancer, emphasizing plant-based precision medicine to augment conventional treatments.

## 1 Introduction

Colon cancer is the fourth most lethal malignancy worldwide. It is estimated that there will be 2 million new cases of colorectal cancer globally in 2020 By 2040, it is projected to reach 3.2 million new cases, with the greatest relative increase occurring in countries in transition and in younger age groups, resulting in a significant burden of disease (1). Despite efforts to lower the screening age and implement early interventions in certain countries, the high fatality rate of colon cancer remains a daunting challenge.

Currently, treatments for colon cancer include surgery, radiotherapy, chemotherapy, immunotherapy, and targeted therapy, however, the efficacy is limited and there are many adverse effects (2, 3). For instance, although chemotherapeutic drugs like 5-Fluorouracil (5-FU) demonstrate evident anticancer efficacy, their detrimental side effects including neurological symptoms, bone marrow suppression, and gastrointestinal reactions intensify patients’ discomfort. Moreover, the development of drug resistance has further compounded the constraints of their clinical utilization (4). Immunotherapy has shown unique advantages in current cancer therapies, especially immune checkpoint inhibitors. The US Food and Drug Administration (FDA) approved anti-PD-1 (Pembrolizumab and Nivolumab) as monotherapy or in combination with cytotoxic T-lymphocyte-associated protein-4 (CTLA-4) inhibitor (Ipilimumab) for the treatment of colon cancer with high microsatellite instability (MSI-H) or mismatch repair deficiency (DMMR), unfortunately, only 5-15% of colon cancer patients benefit (5, 6). New drug development against colon cancer is challenging given the limited efficacy of current treatments and the inevitable off-target effect (7).

Natural products are widely sourced and structurally diverse, with excellent pharmacological activity, demonstrating significant advantages in the treatment of colorectal cancer. Several synthetic derivatives derived from natural products have been used in clinical anti-cancer treatments, such as the semi-synthetic water-soluble camptothecin derivative, irinotecan (8). Many botanical active compounds have shown anti-colon cancer activity, including improving the tumor inflammatory microenvironment, inhibiting tumor cell proliferation, migration, and angiogenesis, as well as regulating the gut microbiota (9–11).

It is known that tumor microenvironment (TME) is critical for cancer progression, of which immune cell subsets are key components. Macrophages, as important members of the immune system, are involved in inflammatory responses and immune regulation, while dynamically changing in response to various signals in TME (12, 13). Studies have shown that in mouse models of colon cancer, macrophages may lose their ability to phagocytose tumor cells and even be used by tumor cells to promote their growth and proliferation (14, 15). Targeted regulation of the activity of macrophages, promotion of its phagocytosis of tumor cells and immunomodulatory function has become a new research hotspot in current cancer therapy. Moreover, because of the stability of the genome of macrophages, they are relatively less likely to develop drug resistance compared to tumor cells (16). Existing reports have found that natural compounds such as Astragaloside, Curcumin can exert anti-colorectal cancer effects by modulating macrophage activity (17, 18). Further investigation into the immune regulatory roles of phytochemicals will help provide more effective treatment options for colon cancer patients. Therefore, this review summarizes the mechanisms by which macrophages are involved in the development of colon cancer and the potential strategies of targeting macrophages with natural products for the treatment of colon cancer.

## 2 Overview of macrophages

### 2.1 Basic biological characteristics of macrophages

Macrophages are innate immune cells of bone marrow and embryonic origin that phagocytose foreign substances or their own components and present them to T cells to activate adaptive immune responses. In addition, macrophages also participate in tissue homeostasis, repair and remodeling by expressing a variety of receptors, cytokines, chemokines, and protein hydrolases (19).

Depending on the tissue-specific microenvironmental signals macrophages acquire different phenotypes, mainly including M1 phenotype (classical activation) and M2 phenotype (alternative activation) (20, 21). M1 macrophages can be activated by bacterial lipopolysaccharide (LPS) and type 1 helper T (Th1) cytokines such as interferon-γ (IFN-γ) and tumor necrosis factor-α (TNF-α), releasing pro-inflammatory cytokines e.g., interleukin(IL)-1β, IL-6, IL-12, and TNF-α, as well as chemokines e.g., C-X-C Motif Chemokine Ligand (CXCL) 9 and CXCL10, and it is capable of presenting antigens via MHC class II molecules (20, 22). M1 macrophage surface markers mainly include CD80, CD86 and CD16/32. M1 macrophages are often considered to have proinflammatory, antitumor, and adaptive immune activation capabilities (23, 24).

**Figure 1:**
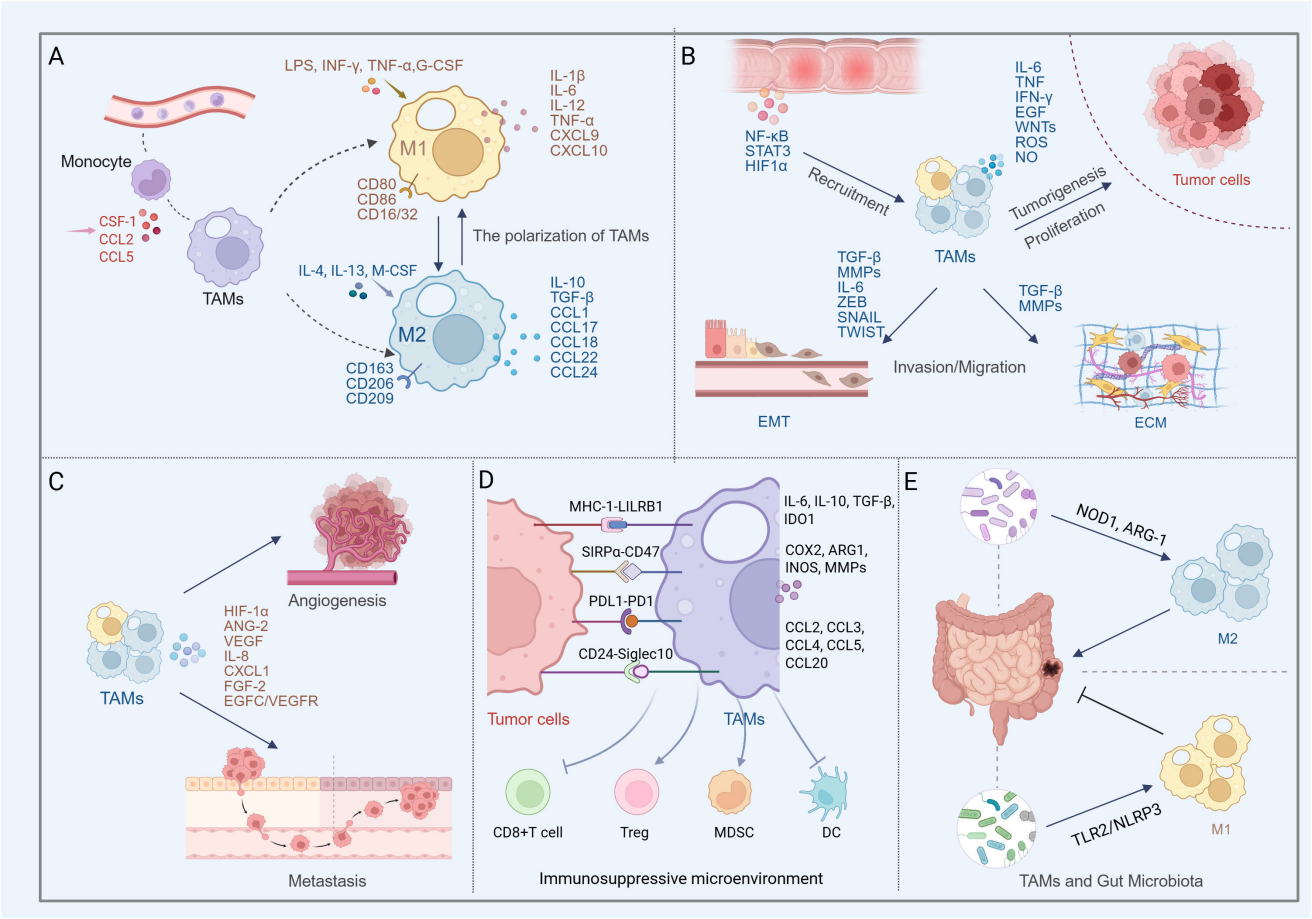
Multifaceted roles of TAMs in colon cancer progression and microenvironment regulation (Created in BioRender. qingman, H. (2025) https://BioRender.com/t5uzu98). **(A)** Peripheral monocytes are recruited by chemokines and differentiate into TAMs. Depending on external stimuli, TAMs polarize into the M1 phenotype, which releases pro-inflammatory factors with anti-tumor and immune-activating functions, or the M2 phenotype, which secretes anti-inflammatory mediators that promote tumor growth and angiogenesis. **(B)** In colon cancer, TAMs predominantly exhibit the M2 phenotype. The TAMs contribute to shaping the inflammatory milieu, promoting EMT, remodeling the ECM, and secreting cytokines, chemokines, and proteases that enhance tumor cell proliferation, invasion, and migration. **(C)** TAM-mediated angiogenesis and metastasis in colon cancer. **(D)** TAMs shape an immunosuppressive microenvironment by secreting inhibitory factors, recruiting Tregs and MDSCs, and suppressing the functions of cytotoxic T cells and dendritic cells. Meanwhile, colon cancer cells evade macrophage-mediated clearance through phagocytosis checkpoints such as CD47-SIRPα, CD24-Siglec-10, MHC-I-LILRB1, and PD1-PD-L1. **(E)** Gut microbiota modulates TAM phenotypes, exerting dual roles in both promoting and suppressing colon cancer progression.

M2 macrophages can be activated by Th2-type cytokines IL-4 and IL-13, producing anti-inflammatory factors such as IL-10 and Transforming growth factor beta (TGF-β), as well as chemokines e.g., C-C Motif Chemokine Ligand (CCL)1, CCL17, CCL18, CCL22, and CCL24, with surface markers CD163, CD206, and CD209 (25). M2 macrophages have the function of promoting the resolution of inflammation and participate in tissue repair through angiogenesis at the later stages of inflammation (26–28). Polarized M2 macrophages are divided into M2a, M2b, M2c and M2d subpopulations. The characteristics of the different subtypes of M2 macrophages are listed in **Table 1**. M2a and M2b types are involved in immune regulation by promoting the Th2 cell response, while the M2c type is associated with immune response suppression and tissue remodeling (43). The M2d type is activated mainly by tumor cells, tumor-associated factors, or chemotherapeutic agents, and is sometimes referred to as TAMs. This type of macrophage has immunosuppressive and tumor-promoting functions (44, 45) and specifically expresses IL-10. and vascular endothelial growth factor (VEGF) to participate in tumor progression and angiogenesis (44, 46) (**Figure 1A**).

**Table 1:** M2 macrophage subtypes and their characteristics.

Phenotype	Stimulating factors	Surface markers	Key secreted molecules	Biological roles	Ref.
M2a	IL-4, IL-13	CD206, CD209, Dectin-1, MHC-II	IL-10, TGF-β, PDGF, IGF, Arg-1, Fizz1, Ym1, CCL17, CCL18, CCL22	induce Th2 response, tissue repair, monocyte recruitment	(27, 29–31; 32)
M2b	Immune complexes + LPS	CD86, MHC-II	IL-10, TNF-α, IL-1β, CCL1	induce Th2 response, mediate pro- and anti-inflammatory response	(33–35)
M2c	IL-10, TGF-β, glucocorticoids, PGE2	CD163, CD206, MerTK	IL-10, TGF-β, Arg-1	promote immunosuppression and clearance of debris	(36–39)
M2d	IL-6, A2R-agonists, TLR-agonists, LPS	CD163	VEGF, IL-10, IL-6, M-CSF, iNOS, CCL2	support tumor progression and angiogenesis	(27, 40–42)

### 2.2 Polarization of tumor-associated macrophages

TME serves as the internal environment in which tumor cells are produced and live and regulates tumor initiation and progression. It consists of non-cancerous cells (a variety of immune cells, cancer-associated fibroblasts, endothelial cells), as well as non-cellular components (cytokines, chemokines, proteins, extracellular vesicles, etc.) (47), and the network formed by these elements builds the tumor-promoting environment (48, 49). Monocytes in the peripheral blood are recruited into the highly structured TME and differentiate into TAMs under the influence of chemokines secreted by tumors, such as colony stimulating factor 1 (CSF-1), CCL2, and CCL5 (50, 51). Significant macrophage accumulation has been found in both tumor mouse models and tumor patient tissue samples (46, 52).

TAMs polarization is usually influenced by complex environmental factors in the TME, such as hypoxia, low pH and variable extracellular matrix (ECM) composition (53, 54). In fact, in the early stage of tumor, the immune system promotes macrophages and T cells to clear tumor cells to play anti-tumor and pro-immune response roles (55), and the majority of infiltrating macrophages at this stage are of the M1 phenotype and secrete pro-inflammatory factors (56). For example, granulocyte colony-stimulating factor (G-CSF) polarizes macrophages to a pro-inflammatory phenotype by promoting IL-10 production and reducing IL-12 in colon cancer TME (57). However, as the tumor progresses and the tumor cells continue to interact with the TME, most TAMs are “educated” to become “bad” macrophages that promote tumor growth and metastasis, and then the TAMs take on a predominantly M2 phenotype, creating an immunosuppressive environment and mediating the Th2 immune response (58). Although the M1/M2 polarization model offers a foundational framework for classifying macrophages, it falls short in capturing the phenotypic diversity and plasticity of TAMs. In recent years, researchers have employed surface markers such as F4/80^+^CD68^+^ and F4/80^+^CD206^+^ to delineate distinct TAM subsets (59). Complementing this approach, clustering analyses derived from single-cell RNA sequencing have identified four novel TAM subpopulations across various solid tumors: FCN1^+^, SPP1^+^, C1Q^+^, and CCL18^+^ macrophages. (60, 61). These advancements contribute to a more nuanced understanding of TAM lineage hierarchies and their functional heterogeneity within the tumor microenvironment. TAMs as one of the abundantly infiltrating immune cells in TME, affect tumor angiogenesis and lymphangiogenesis, tumor cell immune escape, immune regulation, and tumor invasion and metastasis (50, 62) (**Figure 1A**).

## 3 Core roles of TAMs in colon cancer progression

As described in the previous section, TAMs play an important role in the development of tumors. Current studies consider TAMs to be a diverse macrophage population with characteristics of both M1 and M2 subpopulations, rather than being categorized solely by M1/M2 phenotype. In different tumors, the plasticity of macrophages may enable them to play different roles. Several studies have confirmed that macrophage infiltration in the early stage of tumor invasion has a positive impact on the prognosis of colon cancer (63, 64). However, many studies now support the opposite view that TAMs promote malignant progression of colon cancer and play an immunosuppressive role in the TME of colon cancer, as we will describe below.

### 3.1 Promotion of tumor proliferation, invasion, and epithelial-mesenchymal transition

The inflammatory micro-environment involved and mediated by TAMs is closely related to the occurrence and development of colon cancer (65). Activation of cytokines and chemokines produced by chronic inflammation of tissues, as well as transcription factors such as nuclear factor kappa-B (NF-κB), signal transducer and activator of transcription 3 (STAT3), hypoxia-inducible factor 1α (HIF1α), promotes macrophage recruitment and initiates macrophage host responses (66–68).Macrophages subsequently produce inflammatory mediators (IL-6, TNF, IFN-γ), growth factors such as epidermal growth factor (EGF) and WNTs, proteases, reactive oxygen species (ROS), as well as nitric oxide (NO) that may activate proto-oncogenes to promote tissue carcinogenesis (69–71). Not only that, these substances produced by TAMs can also promote colon cancer growth. Studies have found that CCL2, IL-1α and IL-6 secreted by TAMs can promote the proliferation of colon cancer cells (72), and the release of the matrix metalloproteinase (MMP) 1 accelerates the transition of the tumor cell cycle from the G0/G1 phase to the S phase and G2/M phase (73).CSF1 is an important cytokine involved in macrophage proliferation and differentiation, and CSF1 produced by colon cancer cells contributes to the recruitment and infiltration of TAMs. In turn, IL-8 produced by TAMs can activate the PKC pathway on which CSF1 production depends (74).

Epithelial-mesenchymal transition (EMT) is an important development process that is activated during tumor cell migration and invasion (75). Activation of EMT prompts colon tumor cells to lose cell polarity and intercellular adhesion, and to acquire a mesenchymal appearance and greater infiltration and migration capabilities (76). In TME, several EMT-associated transcription factors (ZEB, SNAIL, and TWIST) synergize with signaling pathways in tumor cells (77–79) to induce EMT in response to stimulation by TGF-β, WNT, NOTCH, hypoxia (80–82), etc. TAMs have been demonstrated to have a key role in EMT in colon cancer. M2 macrophages overexpress MMP2 and MMP9, which can induce EMT (83). TGF-β is an important factor in EMT (84). M2 macrophages secrete a large amount of TGF-β, which promotes the expression of mesenchymal marker proteins (Vimentin) and reduces the expression of epithelial marker proteins (E-cadherin) during EMT in colon cancer cells (85). In addition, Wei et al. reported that TAM-derived IL-6 induced the EMT program by regulating the JAK2/STAT3/miR-506-3p/FoxQ1 axis to promote colon cancer cell invasion and migration (86). In colorectal cancer, the number of infiltrating TAMs is positively correlated with the expression of the activating transcription factor SANIL in cancer cells (76). TAMs also promote tumor invasion by up-regulating the expression of S100A8 and S100A9 in cancer cells (87).

Structural support for tumor development is provided by the ECM, including its main components collagen, glycoproteins and proteoglycans (88–90). Afik et al. found that in an orthotopic colorectal cancer model, TAMs promote the deposition, cross-linking, and linearization of collagen fibers to remodel tumor ECM composition and structure and promote tumor development (91).

Colon cancer cells can also activate matrix degradation-related signals to induce TAMs to produce tissue proteases, MMPs and serine proteases, etc., to promote ECM degradation and accelerate the invasion and migration of colon cancer cells (76, 88) (**Figure 1B**).

### 3.2 Mediation of angiogenesis and metastasis

Tumor growth and metastasis require increased intratumoral blood supply, which is usually triggered by tumor hypoxia (92). TAMs infiltrating the TME of colon tumors express HIF1α as an “angiogenic switch”, and respond to hypoxia signals by producing pro-angiogenic cytokines and growth factors, such as Angiopoietin-2 (Ang-2), VEGF, IL-8, CXCL1, and Fibroblast growth factor 2 (FGF-2), to induce endothelial cell recruitment, proliferation, and maturation, forming new blood vessels to promote tumor growth (67, 93, 94). GPR35 on macrophages in colon cancer promotes new angiogenesis and increases MMPs activity through Na/K-ATPase-dependent ion pumps and Src activation (94). Additionally, multiple studies have shown that the number of TAMs in colon cancer correlates with vascular density and the number of lymphatic vessels (95–97). Macrophages on blood vessels emit attractive signals that assist colon cancer cells in migrating along collagen fibers toward the vessels, enabling their escape into the bloodstream. TAMs can also activate the EGFC/VEGFR3 axis, which supports primary colorectal cancer cells growth and metastasis by shaping the lymphatic vessels and providing a pathway for tumor cells to invade the lymphatic system (98) (**Figure 1C**).

### 3.3 Establishment of immunosuppressive microenvironment

During colon cancer progression, the body’s anti-tumor immunity is suppressed, and tumor cells effectively evade anti-tumor immunity by deriving a tumor-immunosuppressive microenvironment, which involves inducing deletion or functional impairment of tumor-reactive T-cells (99) and recruiting immunosuppressive cell populations such as regulatory T-cells (Tregs) and myeloid-derived suppressor cells (MDSCs) (100). TAMs participate in and regulate the tumor immunosuppressive microenvironment by secreting cytokines and altering their phenotype (predominantly M2 macrophages) (101).

TAMs exert immunosuppressive functions through the secretion of inhibitory factors, such as IL-10, TGF-β, and Indoleamine 2,3-Dioxygenase 1 (IDO1) (68, 102), some enzymes, such as COX2, Arginase 1 (ARG1), Inducible nitric oxide synthase (INOS), and MMPs (101), as well as the release of chemokines (e.g., CCL2, CCL3, CCL4, CCL5, and CCL20) (68, 103, 104). The release of these factors not only directly inhibits the activity of T cells (105), but also promotes the recruitment of Tregs and MDSCs in the TME. For example, TAMs promote the aggregation of CCR6+ Treg cells by secreting CCL20 (106), and secreted CCL5 inhibits the killing of HT29 cells by T cells by stabilizing PD-L1, which in turn promotes immune escape (107). In tissue samples from colon cancer patients, the number of Treg cells and macrophages was significantly higher in cancerous tissues than in precancerous tissues (108). The infiltration of a large number of M2 macrophages is associated with poor prognosis, while the infiltration of CD8+ T cells is associated with improved patient survival (109). It was found that cytotoxic T cell responses could be enhanced to prevent colon cancer metastasis by inhibiting TGF-β produced by M2 macrophages (110). In addition, infiltration of high-density CXCR6^+^ TAMs promote the overexpression of IL-6, which would inhibit the activation of CD8^+^ T cells, thus contributing to immunosuppression (111). Ding et al. demonstrated that targeted inhibition of MDSCs could improve the immune microenvironment of colon cancer, not only promoting the maturation of dendritic cells and tumor infiltration of CD8^+^ T cells, but also reducing the number of Tregs and M2 macrophages (112) (**Figure 1D**).

### 3.4 Phagocytosis checkpoints and tumor immune evasion

Macrophages, as “professional” phagocytes in the innate immune system, play the role of scavengers. However, to evade clearance, tumor cells usually overexpress anti-phagocytic membrane proteins with “don’t eat me” signals, such as CD47, CD24, MHC-I, and PD-L1. These antiphagocytic proteins may interact with receptors on macrophages (e.g., SIRPα, Siglec-10, LILRB1, and PD-1) to evade phagocytosis. The anti-phagocytic signals generated by these binding are known as “Phagocytosis checkpoints”. Targeting phagocytosis checkpoints such as CD47-SIRPα, CD24-Siglec-10, MHC-LILRB1, PD1-PDL1 and thereby modulating immune escape in colon cancer cells has also been demonstrated.

Studies have found that using blocking antibodies targeting CD47 overexpressed on colon cancer cells and SIRPα on TAMs can inhibit the migration of colon cancer cells (113–115). In addition, phagocytic activity of macrophages was enhanced after anti-SIRPα antibody treatment (116). Interestingly, under hypoxic conditions, TAMs showed decreased SIRPα expression accompanied by increased phagocytic activity, whereas colon cancer cells showed elevated expression of CD47 and exhibited greater invasiveness (117). Therefore, it can be hypothesized that the regulation of the CD47-SIRPα axis in colon cancer may be affected by hypoxia.

CD24 serves as a critical “don’t eat me” signal molecule expressed by tumor cells. Several studies have indicated that its overexpression in colorectal cancer cells is associated with early-stage tumor metastasis (118). In colon cancer xenograft mouse models, treatment with anti-CD24 antibodies effectively suppressed the tumor growth (119, 120). Interestingly, Nersisyan et al. reported that reduced CD24 expression or gene loss in colon cancer patients was linked to shorter overall survival (121). These findings suggest that CD24 may function as a stage-specific driver during colorectal tumorigenesis—upregulated in early disease and potentially regulated later by shifts in immune cell activity. Zhao et al. further found a negative correlation between CD24 expression and multiple immune-related pathways, including TNF-α, NF-κB, and IFN-γ signaling (122). Siglec-10 has also been identified as an early driver gene in colorectal malignancies, with mutations observed in both multiple colonic adenomas and colorectal tumors (123). Single-cell RNA sequencing data revealed an enrichment of FOLR2^+^ LYVE1^+^ macrophages in KRAS/TP53 double-mutant colon cancer, where tumor cells appear to engage in immunosuppressive crosstalk with macrophages via the CD24–Siglec-10 axis. (124). Blocking the CD24–Siglec-10 anti-phagocytic axis effectively activates macrophage-mediated phagocytosis in the MC38 colon cancer model (125).

In colorectal cancer DLD1 cells, MHC-I expression suppresses macrophage-mediated phagocytosis and promotes immune evasion, primarily through its interaction with LILRB1 on TAMs. *In vivo* studies have shown that CRISPR-mediated knockout of MHC-I molecules in tumor cells suppresses tumor growth in a macrophage-dependent manner (126). However, in tumor tissues from patients with colon cancer liver metastases, high expression of MHC-I (β2m) on tumor cells has been associated with improved overall survival and increased T cell infiltration (126). The dual role of MHC-I in tumor immune evasion may stem from its capacity to engage both innate and adaptive immune responses. When functioning as an antigen-presenting molecule to cytotoxic T lymphocytes, MHC-I may no longer interact effectively with LILRB1. Additionally, some studies have reported that reduced MHC expression on tumor cell surfaces impairs antigen presentation, which may, in turn, enhance macrophage-mediated phagocytosis (127).

PD-1/PD-L1 inhibitors have been shown to effectively reverse T cell immune tolerance. In addition to T cells, PD-1 is also expressed on other immune cells, including macrophages, B cells, NK cells, and dendritic cells. Notably, increasing attention has been directed toward the functional role of PD-1/PD-L1 signaling in TAMs (128). Studies have reported that colon cancers with MSI-H tend to exhibit elevated PD-L1 expression, and that PD-1 levels on TAMs increase as the disease progresses (14, 129). PD-1 expression on macrophages has been implicated in promoting polarization toward the immunosuppressive M2 phenotype. In murine models of colorectal cancer, PD-1 blockade has been shown to reduce the infiltration of M2 macrophages within the TME (130). The PD-1/PD-L1 axis is considered part of an anti-phagocytic signaling pathway. In both colon cancer patients and mouse models, high PD-1 expression on TAMs has been associated with reduced phagocytic activity against tumor cells. Disruption of this axis can induce M1 polarization of macrophages and promote CD8^+^ T cell activation, thereby enhancing antitumor immunity (14). (**Figure 1D**).

### 3.5 The metabolic plasticity of TAMs

In pro-inflammatory or anti-inflammatory TME, TAMs exhibit notable metabolic plasticity. They comprehensively reprogram their energy sources, metabolic pathways, and metabolites to adapt to tumor progression. In colon cancer, tumor cells undergoing rapid proliferation produce lactate through the Warburg effect. The resulting acidic environment facilitates TAM recruitment and induces their immunosuppressive functions.

Similar to tumor cells, M1 macrophages rely on aerobic glycolysis and the pentose phosphate pathway (PPP), with upregulation of HIF-1α-related signaling, promoting the production of lactate and succinate (131). In contrast, M2 macrophages exhibit lower glucose consumption and primarily depend on fatty acid oxidation (FAO) and oxidative phosphorylation (OXPHOS) for energy supply. They express high levels of CD36, facilitating fatty acid uptake and β-oxidation. Additionally, FAO-induced mitochondrial stress and elevated ROS levels contribute to M2 polarization (132). It has been shown that the STING agonist GB2 can induce metabolic reprogramming of TAMs in colorectal cancer by upregulating the glycolysis–ROS–HIF-1α axis and downregulating OXPHOS, thereby promoting M1 polarization and enhancing phagocytic activity (133).

Increased glucose uptake leads to the accumulation of lactate in the TME. Zhang et al. were the first to reveal the role of histone lysine lactylation in the modification of macrophages, demonstrating that the level of lysine lactylation is positively correlated with intracellular lactate concentration (54). In colon cancer, elevated lactate levels promote M2 polarization and the secretion of signaling molecules that facilitate tumor progression. (76, 133). For example, lactate in the tumor microenvironment epigenetically upregulates the expression of VSIG4 in macrophages, which subsequently activates the JAK2/STAT3 pathway, enhances fatty acid oxidation and PPAR-γ expression, and drives polarization toward an immunosuppressive M2 phenotype (134).

In terms of amino acid metabolism, M1 macrophages primarily utilize arginine metabolism by upregulating iNOS to produce citrulline and NO, thereby enhancing antimicrobial and antitumor activity. In contrast, M2 macrophages upregulate ARG1, converting arginine into ornithine and polyamines, which contribute to immunosuppression (16). Increased expression of ARG1 has been identified as a key factor in rapid tumor growth and progression (16). Additionally, M2 macrophages metabolize tryptophan via IDO1 to generate kynurenine, which suppresses T cell function and promotes immune tolerance. It has been shown that inhibiting ARG1 while activating iNOS can effectively increase the number of M1 macrophages and suppress colon tumor growth (135).

### 3.6 Exosomal crosstalk with colon cancer cells

**Figure 2:**
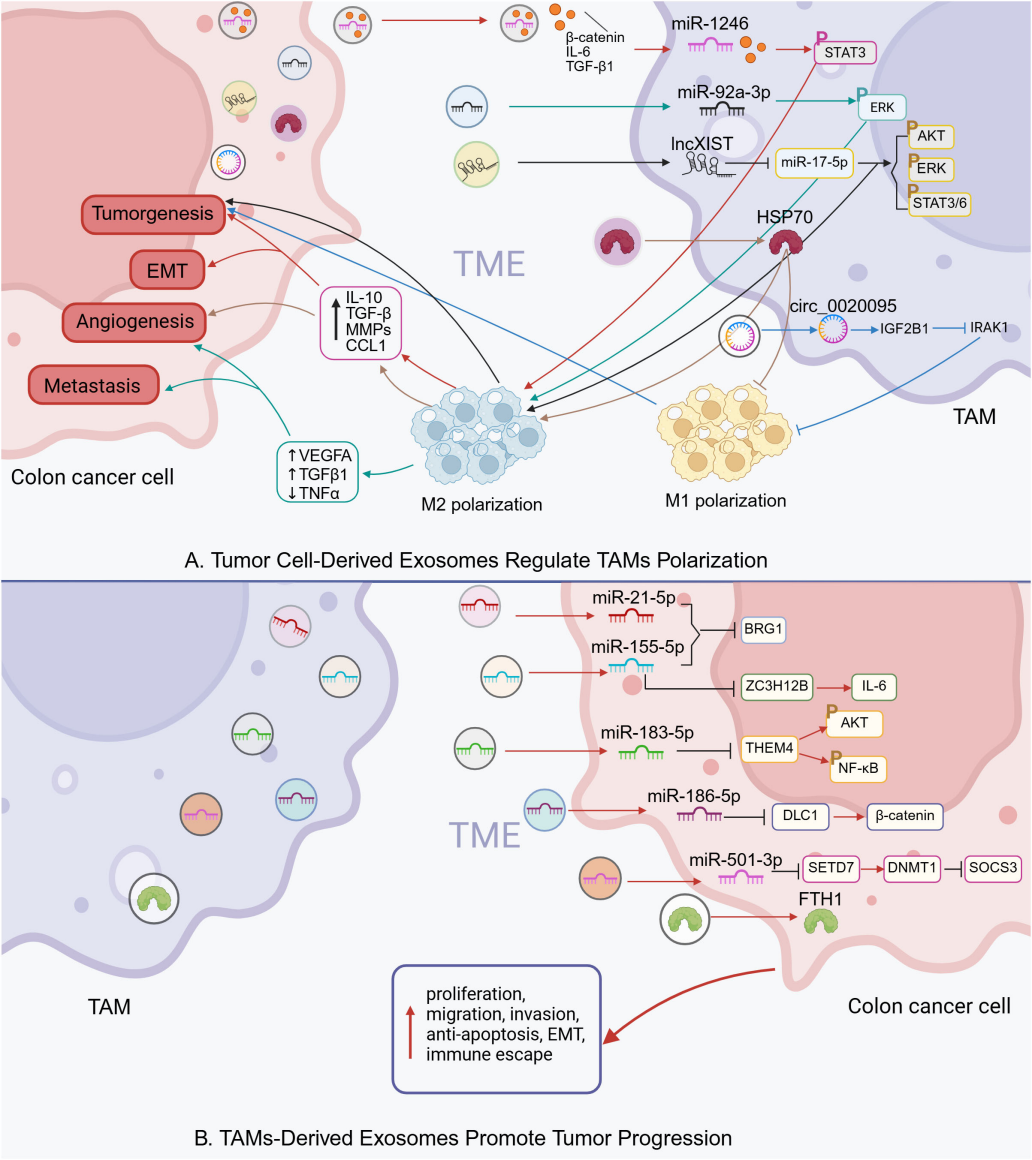
Intercellular communication via exosomes in colon cancer TME (Created in BioRender. qingman, H. (2025) https://BioRender.com/ijd8zht). **(A)** Tumor cell-derived exosomes regulate TAMs polarization. **(B)** TAMs-derived exosomes promote tumor progression.

Autocrine and paracrine signaling mediated by cytokines and chemokines plays a pivotal role in the communication between tumor cells and TAMs. Both tumor cells and TAMs are capable of internalizing exosomes secreted by one another, thereby releasing their endosomal contents. This exchange contributes to the regulation of macrophage polarization and influences key tumor behaviors, including invasion, metastasis, and immune evasion (**Table 2**) (**Figure 2**).

**Table 2:** Regulation of tumor immunity by TAMs and colon cancer cell-derived exosomes.

Exosomes type	Cargos	Name	Mechanism	Biological function	Ref.
Tumor cells–derived	miRNA	miR-1246	↑ TGF-β, IL-10, MMP	promote M2 polarization, EMT, tumorgenesis	(136)
			↑ β-catenin, IL-6, p-STAT3, TGF-β1	promote M2 polarization	(137)
	miRNA	miR-92a-3p	↑ p-ERK, TGF-β, VEGFA, ↓ TNF-α	promote M2 polarization, tumor cell invasion, migration, angiogenesis	(138)
	circRNA	circ_0020095	↑ IGF2BP1, ↓ IRAK1	inhibit M1 polarization, promote tumor cell proliferation, invasion, migration	(139)
	lncRNA	lncXIST	↓ miR-17-5p,↑ PDGFRA, ↑ p-AKT, p-ERK, p-STAT3, p-STAT6	promote M2 polarization, tumor cell proliferation, invasion, migration	(140)
	protein	HSP90B1	↓ TNF-α, IL-6, IL-12, ↑ IL-10, TGF-β, CCL-1	promote M2 polarization, inhibit CD8+ T cell viability, promote liver metastasis	(141)
	protein	HSP70	↓ IL-10, ↑ TNF-α, IFN-γ	promote immune response, CD8+ T cell infiltration	(142)
TAMs-derived	miRNA	miR-21-5p	↓ BRG1	promote tumor cell invasion, migration	(143)
	miRNA	miR-155-5p	↓ BRG1	promote tumor cell invasion, migration	(143)
			↑ IL-6, ↓ ZC3H12B	promote tumor cell proliferation, anti-apoptosis, immune escape, inhibit CD3+ T cell proliferation	(144)
	miRNA	miR-183-5p	↓ THEM4, ↑ p-AKT, p-NF-κB	promote tumor cell proliferation, invasion, migration, anti-apoptosis	(145)
	miRNA	miR-186-5p	↓ DLC1, ↑ β-catenin	promote tumor cell proliferation, invasion, migration, EMT	(146)
	miRNA	miR-501-3p	↓ SETD7, ↑ DNMT1, ↓ SOCS3	promote tumor cell invasion, migration	(147)
	protein	FTH1	/	promote tumor cell proliferation	(148)

In colon cancer, exosomes released by TAMs and colon cancer cells jointly contribute to the regulation of tumor immunity. Specifically, colon cancer cells can secrete exosomes containing various non-coding RNAs—including microRNAs (miRNAs), long non-coding RNAs, and circular RNAs, which influence TAM polarization. For instance, exosomal miR-1246 and miR-92a-3p derived from tumor cells have been shown to activate STAT3 and ERK signaling pathways in TAMs, promoting their polarization toward an immunosuppressive M2 phenotype (137, 149). Similarly, circ_0020095 facilitates tumor cell invasion and migration by downregulating IRAK1 expression and suppressing M1 polarization (139). In addition to RNA cargo, tumor-derived exosomes can also transport specific proteins that modulate the immune response. HSP90B1, delivered via exosomes from colorectal cancer cells, has been associated with poor prognosis in advanced colorectal cancer and contributes to liver metastasis by promoting the conversion of M1 to M2 macrophages (141). Notably, treatment targeting HSP70-positive exosomes produced by colorectal cancer cells has been reported to enhance antitumor immune responses, suggesting that TAMs possess functional plasticity (142).

On the other hand, TAMs can also influence tumor progression through the transfer of RNA and protein molecules encapsulated within exosomes. For example, M2 macrophages have been shown to promote colon cancer cell proliferation by delivering ferritin heavy chain (FTH1) protein via exosomes (148). Additionally, TAM-derived exosomes exhibit high levels of miR-21-5p and miR-155-5p, which target and suppress the expression of BRG1 in tumor cells, thereby enhancing their malignant properties and facilitating immune evasion (143).

### 3.7 Interaction with gut microbiota

Evidence suggests that chronic low-grade inflammation due to gut microbial dysbiosis as well as impaired intestinal barriers are important factors driving colon cancer development (150–152). Infiltrating bacterial products can construct an immunosuppressive TME by shaping TAMs (153). Fewer macrophages were found in the intestinal wall of germ-free mice compared to SPF mice (154, 155). Enrichment of Fusobacterium nucleatum, Bacteroides fragilis, Escherichia coli, and Enterococcus faecalis has been observed in the feces or tumor tissues of colorectal cancer patients (156–159). The establishment of chronic inflammation promotes colorectal cancer development. In APCmin/+ mice, Fusobacterium nucleatum facilitates the recruitment and infiltration of specific myeloid cell subsets into tumor tissue, producing NF-kB-related pro-inflammatory characteristics (160, 161). In a mouse model infected with Citrobacter rodentium, macrophage-derived ornithine decarboxylase promotes M1 macrophages activation, leading to increased mucosal inflammation in the colon (162). Gut microbes also promote colon carcinogenesis by modulating macrophage immunoreactivity. In Fusobacterium nucleatum-fed mouse models with intestinal tumors, M2-like macrophage accumulation has been observed within tumor tissues, showing significant suppressive activity against CD4+ T cells (160). Additionally, bacterial products such as muramyl peptides activate NOD1, maintaining ARG-1 overexpression and promoting colorectal tumor formation by modulating the immunosuppressive activity of MDSCs and TAMs (153). However, most current studies have focused on the crosstalk between tumorigenic bacteria and TAMs, and the role of some beneficial bacterial species is easily overlooked. For example, Akkermansia muciniphila, a probiotic with strong adhesion to the intestinal epithelial cell surface, can mediate M1 macrophages accumulation in a TLR2/NLRP3-dependent manner to inhibit colorectal tumorigenesis (163) (**Figure 1E**).

## 4 Therapeutic strategies targeting TAMs in colon cancer

**Figure 3:**
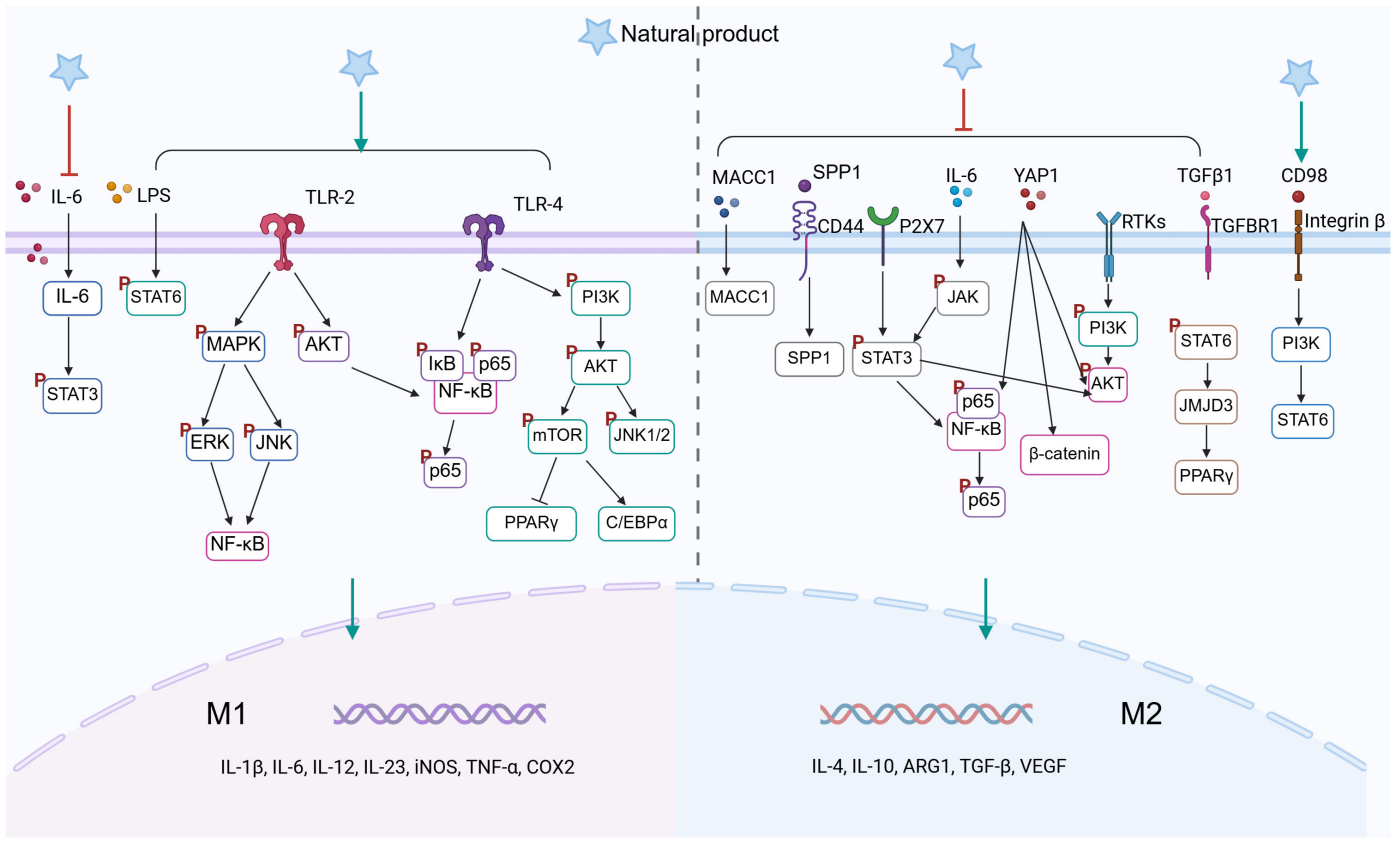
Natural product–mediated signaling pathways in TAMs polarization (Created in BioRender. qingman, H. (2025) https://BioRender.com/3g8lx62).

Historically, natural products have provided many active molecules with novel structures for new drug development. Many anticancer compounds derived from natural products or their derivatives, such as paclitaxel and vincristine, have played an important role in the clinical treatment of cancer. Recent preclinical studies have further confirmed that natural products can effectively prevent and treat colon cancer by targeting TAMs. We summarize various natural products, including monomeric compounds, synthetic formulations based on natural products, combination therapies of natural products with existing anti-cancer drugs, nanoparticle-based drugs, traditional Chinese medicine, and dietary interventions. The primary mechanisms by which these natural products target TAMs include: inhibition of the inflammatory tumor microenvironment, regulation of macrophage polarization (such as inducing M1 polarization and suppressing M2 polarization) (**Figure 3**), modulation of metabolism, enhancement of macrophage phagocytic function, and modulation of other immune cells through TAMs, thereby effectively inhibiting immunosuppressive TME. These aspects will be discussed in detail in the following sections.

### 4.1 Botanical compounds and medicinal plants

Studies have confirmed that botanical compounds exhibit significant pharmacological and biological activities in the treatment of colon cancer (164, 165). Among these, polysaccharides, polyphenols, saponins, quinones, and terpenoids have been found to exert anti-tumor effects by modulating TAMs. The specific mechanisms of action are summarized in the table below (**Table 3**).

**Table 3:** Natural products targeting TAMs for colon treatment and the mechanism of action.

Categories	Compound	Sources	Pharmacological function	*In vivo* model and administration dose	*In vitro* model	Molecular mechanisms	Effect on the macrophages	Anti-tumor function	Ref.
Polyphenols	Procyanidins	Litchi	anti-inflammation, anticancer, antioxidation	female BALB/c mice: CT26 tail vein injection	/	↓ CD68, ARG1, CD206, CCL2	decrease TAMs infiltration	suppress tumor growth and lung metastasis, activate T cell immunity, alleviate macrophages induced inflammation	(166)
	Curcumin	Curcumae longae Rhizoma	anticancer, antioxidation, immune modulation	/	THP-1 SW620, HCT116	↓ IL-10, ARG1, CD163, MACC1	inhibit M2 polarization	suppress tumor cell proliferation and migration	(167)
	Piceatannol	seeds of Euphorbia lagascae	anti-inflammation, anticancer, antioxidation	male BALB/c athymic nude mice: SW480 and M2-like macrophages subcutaneous injection, 10, 50 mg/kg	THP-1 SW480	↓ CD163, TGF-β1, ARG1, Fibronectin	inhibit M2 polarization	suppress tumor growth and EMT	(168)
	Luteolin	ResedaodorataL.	anti-inflammation, anticancer, antioxidation	/	THP-1 SW480, SW620	↓ IL-6, p-STAT3	inhibit M1 polarization	suppress tumor cell proliferation and invasion	(169)
	Cannabidiol	Cannabis	neuroprotective, anti-inflammation, anticancer, anti-emetic	C57BL/6J: MC38 subcutaneous injection, 10 mg/kg	BMDM MC38	↓ CD206, ↑ CD86, iNOS, ↓ ATP, MDA, OXPHOS, β-oxidation, p-P13K, p-AKT, ↑ glycolysis	promote M1 polarization, inhibit M2 polarization	suppress tumor growth, modulate the suppressive TME, reduce Tregs and enhance CD8+ T and NK cell infiltration	(170)
	Polyphenols-rich components from seaweeds	Seaweeds,Gracilaria	anti-inflammation, anticancer	/	RAW264.7 CCD-18Co, HCT116	↓ NO, IL-1, IL-6, TNF-α, iNOS, COX2, ↑ HO-1	regulate TAMs pro-inflammatory activity	suppress tumor cell proliferation, promote apoptosis and cell cycle arrest	(171)
	Non-extractable phenolics from strawberry	Strawberry	anti-inflammation, anticancer, anti-diabetes	/	RAW264.7 HCT116	↓ iNOS, c-FOS, ↑ HO-1	reduce TAMs inflammation	suppress tumor cell proliferation, migration, induce cell cycle arrest in G2/M phase, promote apoptosis	(172)
	Hydroxygenkwanin	Dracocephalum moldavica L.	anticancer, anti-inflammation, antioxidation	C57BL/6J: MC38 intraperitoneally injection, 0.1, 0.25, 0.5 mg/kg	BMDM MC38	↑ TNF-α, iNOS, IL-6, IL-1β, p-STAT5, p-NF-κB, ↓ARG1, CD206, Ym1, JMJD3, p-STAT6, PPAR-γ	promote M1 polarization, inhibit M2 polarization	suppress tumor growth and peritoneal metastasis through the modulation of macrophages	(173)
	Tetrahydrocurcumin	Curcuma longa L.	anticancer, anti-inflammation, antioxidative	AOM/DSS mice: 50, 100, 200 mg/kg	THP-1 HCT116, MC38	↓ CD206, ARG1, IL-10, SPP1, ↑ iNOS, IL-1β, TNF-α	inhibit M2 polarization	suppress tumorgenesis and progression via modulating the SPP1/CD44 axis and activating ERK signaling pathway	(174)
Polysaccharides	Fucoidan	Brown algae	anticancer, immune modulation, antioxidation	male BALB/C mice: HCT116 subcutaneous injection, 200, 500 mg/kg	RAW264.7 HCT116, RKO	↑ CD86, ↓ CD206, ↑ TLR4, p-P13K, p-AKT, p-mTOR, C/EBPα, glycolysis, ↓PPAR-γ	promote M1 polarization	inhibit tumor growth, promote tumor cell apoptosis	(175)
	Polysaccharides from Nostoc commune	Nostoc commune Vaucher	antioxidation, anti-inflammation, anti- carcinogenesis	BALB/c mice: CT26 xenograft, 200 mg/kg	BMDM SW480, SW620, DLD-1, CT26, HT-29, HCT116	↑ TNF-α, IL-1β, IL-6, IL-12β, p-AKT, p-JNK1/2, ↓ IKB-α	promote M1 polarization	inhibit tumor growth, promote tumor cell apoptosis	(176)
	Safflower polysaccharide	Safflower petals	anti-inflammation, anticancer, immune modulation	AOM/DSS mice: 50 mg/kg	RAW264.7 HCT116, SW480, LoVo, RKO, MC38	↑ iNOS, IL-23, CD16, CD80, p-p65, p-IKBα, TNF-α, IL-6, NO	promote M1 polarization	suppress tumor growth and induce tumor cell apoptosis via regulating macrophage polarization	(177)
	Cnidium officinale polysaccharide	Cnidium officinale	anti-inflammation, anti-anemia	/	RAW264.7 HCT116	↑NO, TNF-α, IL-1β, IL-6, IL-10, p-p65, p-p38, p-JNK, p-ERK1/2	enhance pro-inflammatory activity of macrophages	promote tumor immune response	(178)
	Beta-1,6 glucan	Armillaria mellea	anti-inflammation, anticancer, immune modulation, antioxidation	female BALB/c mice: CT26 subcutaneous injection, 10, 20 mg/kg	RAW264.7 CT26, DLD-1, HCT116	↑ NO, TNF-α, IL-6,iNOS, ↓ IL-10, CD206, Ym1, ARG1, IKBα, ↑ p-p65, p-ERK, p-JNK	promote the polarization of M2 to M1	suppress tumor growth, promote tumor cell apoptosis	(179)
	Ganoderma lucidum polysaccharide	Ganoderma lucidum	anti-inflammation, anticancer, immune modulation	APCmin/+ mice: 750 mg/kg	/	↓ IL-1β, IFN-γ, FOXP3, TNF-α, ↑ IL-4, IL-10, IL-12, IL-13	promote the polarization of M1 to M2	reduce polyps, prevent colon tumorigenesis, regulate gut microbiota	(180)
	Polysaccharides from ginseng leaves	Ginseng leaves	anticancer, immune modulation	BALB/c mice: colon 26-M3.1 tail vein injection, 4-500 μg/mice	peritoneal exudate macrophages Colon 26-M3.1	↑ TNF-α, IL-12	activate macrophage	suppress tumor growth and metastasis	(181)
Saponins	panaxynol	American ginseng	anti-inflammation, immune modulation, antioxidation	AOM/DSS mice: 2.5 mg/kg	BMDM	↓ IL-6, IL-13, TGF-β1	decrease M2 infiltration	suppress tumor growth, promote tumor cell apoptosis	(182)
	Ginsenoside-Rp1	Panax ginseng	anti-inflammation, immune modulation, antioxidation	/	J774A.1 CT26	↓ NO, IL-1β	inhibit TAMSs activation	suppress tumor cell proliferation	(183)
	Astragaloside IV	Astragalus membranaceus	anti-inflammation, antioxidation, lowering blood pressure	female BALB/C mice: CT26 subcutaneous injection, 15 mg/kg	BMDM CT26	↓ ARG1,Mrc1, TGF-β, IL-10, VEGF-α, ↑ IL-12, Nos2, IFN-γ, TNF-α	promote the polarization of M2 to M1	suppress tumor growth and activate immune response	(184)
	Gypenoside	Gynostemma pentaphyllum	anti-inflammation, antioxidation, metabolic Regulation	APCmin/+ mice: 300 mg/kg	/	↓ iNOS↓, CXCL10, ↑ Ym-1, CD206, Trem2, ARG1	promote the polarization of M1 to M2	reduce polyps, prevent colon tumorigenesis	(180)
Terpenoids	Diterpene ent-polyalthic acid	Copaifera reticulata Ducke oleoresin	analgesic, antioxidation, anticancer	male Wistar rats: 1,2-dimethylhydrazine intraperitoneal injection, 80 mg/kg	peritoneal macrophages	↓ NO, PGE2	inhibit TAMs infiltration	suppress colon carcinogenesis and DNA damage	(185)
	dihydroisotanshinone I	Salvia miltiorrhiza Bunge	anticancer, cardioprotective effects	BALB/c-nu nude mice: HCT116 xenograft, 30 mg/kg	RAW264.7 HCT116, HT-29	CCL2↓	suppress TAMs activation	suppress tumor cell recruitment ability of macrophage, promote tumor cell apoptosis	(186)
	Triptolide	Tripterygium wilfordii Hook. F	anti-inflammation, antimicrobial, antifibrosis, anticancer	BALB/c mice: CT26 subcutaneous injection, 0.1, 0.2, 0.4 mg/kg	RAW264.7 HT29, CT116	↓ ARG1, CD206, IL-10, CXCL12	decrease TAMs infiltration, inhibit M2 polarization	suppress tumor growth and remodel TME through NF‐κB p65 and ERK1/2 axis	(187)
	Oridonin	Rabdosia rubescens	anticancer, anti-angiogenesis	male BALB/c nude mice: HCT116 subcutaneous injection, 40 mg/kg	/	↓ CD206	decrease M2 infiltration	suppress tumor growth and angiogenesis through JAK2/STAT3 signaling pathway	(188)
	Cucurbitacin B	Muskmelon pedicel	anticancer, anti-inflammation, antioxidation	C57BL/6 mice: CT26 subcutaneous injection, 0.5, 1 mg/kg	RAW264.7, THP-1 HCT116, CT26	↓ Ym1, Fizz1, ARG1, CCR2, p-JAK, p-STAT3	inhibit M2 polarization	suppress tumor growth, inhibit tumor cell invasion and migration, regulate TME	(H. 189)
	Oleuropein	Olea europaea L. leaves	anti-inflammation, immune modulation, antioxidation	PIRC rats: 100 mg/kg	RAW264.7 HCT116	↓ iNOS, IL-1β, IL-6, TGF-β, COX2	suppress TAMs activation	suppress tumorgenesis, promote tumor cell apoptosis	(190)
Quinones	Emodin	Rheum palmatum, Polygonum cuspidatum, and polygonum multiflorum	anti-inflammation, anticancer	APCmin/+ and AOM/DSS mice: 40, 80 mg/kg	BMDM C26	↓ P2X7, p-STAT3	decrease M2 proliferation	reduce polyp and tumorgenesis, regulate TME	(191)
	Bullatacin	Annonaceae	anticancer	/	SW480, HT-29	↑ CRT, HSP90, HMGB1	promote macrophage phagocytosis	promote immunogenic tumor cell death by activating endoplasmic reticulum stress	(192)

#### 4.1.1 Polyphenols: remodeling immune microenvironment and inhibiting inflammation

Polyphenols are the most common plant-based bioactive constituents of the diet and are widely found in fruits, vegetables, cereals, coffee and green tea. Numerous experimental studies have demonstrated that polyphenols (e.g., procyanidins (166), curcumin (167), piceatannol (168), luteolin (169)) have significant anti-inflammatory, anti-tumor, pro-apoptotic, and immunomodulatory effects in colon cancer. Mechanistic studies indicate that these effects are primarily achieved through the regulation of TAM polarization, downregulation of inflammatory signaling, enhancement of immune responses, modulation of macrophage metabolism, and regulation of the gut microbiota. For example, single-cell sequencing analysis has shown that cannabidiol, a non-psychoactive compound from cannabis, promotes TAM glycolysis while inhibiting FAO and oxidative phosphorylation OXPHOS. It also regulates the PI3K/AKT pathway, promoting M1 polarization of TAMs to exert anti-colon cancer effects (170). Meanwhile, some polyphenol-rich extracts/non-extractable polyphenolic substances are also of interest. For example, polyphenol-rich components from edible seaweeds (171) and non-extractable phenolic from strawberries (172) were able to inhibit the pro-inflammatory activity of LPS-stimulated macrophages, induce cell cycle arrest in HCT116 human colon cancer cells, and promote their apoptosis.

#### 4.1.2 Polysaccharides: activating M1 polarization and modulating signaling pathways

Natural polysaccharides have a wide range of biological activities, with anti-tumor, antioxidant, liver protection, immunomodulation and other functions. Because of their good therapeutic effects and mild adverse reactions, they have received extensive attention in the field of biomedicine. Many natural polysaccharides, such as fucoidan, safflower polysaccharides, cnidium officinale polysaccharides, and ganoderma lucidum polysaccharide, are effective in preventing and controlling colon cancer by regulating the signaling pathway, influencing the macrophage phenotype and function, enhancing immune response, and maintaining intestinal homeostasis.

Polysaccharides from algae have been extensively studied, for example, fucoidan extracted from brown algae activates the TLR4-mediated PI3K/AKT/mTOR signaling axis, promoting M1 polarization and infiltration of TAMs, thereby altering the immunosuppressive TME (175). Polysaccharides from Nostoc commune Vaucher can inhibit colon cancer cell proliferation by activating NF-κB, AKT/JNK1/2 signaling pathway, upregulating the expression of inflammatory factors, and enhancing the phagocytic activity of TAMs (176). Studies have shown that safflower polysaccharide (108), Cnidium officinale polysaccharide (178) and Beta-1,6 glucan (179) can activate the NF-κB and MAPK signaling pathways in colon cancer, promote the activation of M1 macrophages in the TME to generate immune responses. Shin et al. found that treatment of colon cancer model mice with the polysaccharides from ginseng leaves exhibited antitumor activity, and it promoted the secretion of TNF-α and IL-12 from macrophages in peritoneal exudates (181). Adenomatous polyps are considered precancerous lesions of sporadic colon cancer, and Ganoderma lucidum polysaccharide were effective in improving the inflammatory intestinal barrier in APCmin/+ mice reducing the formation of adenomatous polyps by decreasing the infiltration of inflammatory TAMs (180).

#### 4.1.3 Saponins and terpenoids: modulation TAMs recruitment and suppressing M2 polarization

Studies on the regulation of TAMs in colon cancer by saponin compounds are mainly focused on ginsenosides. Ginsenosides have a variety of pharmacological activities and biological effects, such as anti-inflammatory, antioxidant, immunomodulatory, anti-tumor, and pro-apoptotic. Panaxynol, isolated from ginsenosides, promotes tumor cell apoptosis by effectively inhibiting M2 polarization in colon cancer TME (182). Furthermore, ginsenoside-Rp1 inhibits phenotypic variation in CT26 colon cancer cells by suppressing LPS-stimulated macrophage activation (183). Astragaloside IV (184) and jiaogulan saponins (180) extracted from traditional Chinese medicine, were able to reduce the formation of intestinal polyps and tumors by modulating the ratio of pro-inflammatory to anti-inflammatory macrophages in the TME.

From the current reports, it has been found that terpenoids improve colon cancer outcomes by interfering with TAMs including 1) prevention of colon precancerous lesions, 2) inhibition of tumor cell recruitment, proliferation, migration, and angiogenesis, 3) promotion of apoptosis, and 4) activation of immune response. Copaifera reticulata Ducke oleoresin, widely used in Brazilian folk medicine, has wound healing-promoting and anti-inflammatory effects. Its main chemical component, diterpene ent-polyalthic acid, reduces DNA damage and preneoplastic lesions induced by the carcinogen, also reduces NO and PGE2 production in mouse TAMs, demonstrating the potential for preventing the development of colon cancer (185). Dihydroisotanshinone I, a bioactive compound in Salvia miltiorrhiza Bunge can hinder the recruitment of TAMs to colon cancer cells by inhibiting the secretion of CCL2 from TAMs (186). Jiang et al. found that tretinoin inhibited NF-κB and ERK1/2 signaling and reduced M2 macrophage polarization remodeling TME by downregulating CXCL12 expression (187). Oridonin has excellent anti-angiogenic effects in colon cancer, reduces M2 macrophage infiltration, and also improves the hypoxic environment (188).

#### 4.1.4 Quinones: inducing immunogenic cell death

Quinones are widely distributed in nature and are categorized into four major subclasses, including benzoquinone, naphthoquinone, phenanthrenequinone and anthraquinone. There are limited reports on whether quinones can prevent colon cancer by affecting TAMs, which have only been reported in Bullatacin. Emodin is a naturally occurring anthraquinone compound isolated from herbs such as rhubarb, Tigen stick as well as Tuber Fleeceflower Root, and has a variety of biological activities including anti-inflammatory, antioxidant, and antitumor. Studies have confirmed the role of Emodin in regulating colonic TME, not only by decreasing the proportion of M2 macrophages, but also by reducing inflammatory activation through inhibition of the P2X7 receptor (191). Bullatacin, a natural quinone compound isolated from the plant Lycopersiconaceae, induces immunogenic cell death (ICD) in tumor cells. Fan et al. demonstrated that treatment with Bullatacin promotes the accumulation of the “eat me” signaling proteins, CRT and HSP90, on the surface of colorectal tumor cells. It triggers ICD by activating the endoplasmic reticulum stress (ERS) signaling pathway and promotes phagocytosis of tumor cells by macrophages (192).

### 4.2 Natural product–based synthetic agents

Natural compounds face limitations in clinical applications, such as unstable efficacy, poor solubility, and challenges in standardization. To overcome these issues, synthetic modifications can be employed to create novel drug formulations by knocking out or altering certain unstable structures of the compounds. We summarize the role and mechanisms of existing synthetic formulations based on natural products targeting TAMs in colon cancer (**Table 4**).

**Table 4:** Synthetic agents derived from natural products and combination strategies targeting TAMs in colon cancer.

Drug	Pharmacological function	Combination agent	*In vivo* model and administration dose	*In vitro* model	Effect on macrophages	Anti-tumor function	Ref.
Aspirin	antiplatelet, anti-inflammation, antipyretic	**/**	AOM/DSS, AOM mice: 25, 50 mg/kg	**/**	↓ macrophages infiltration, iNOS	inhibit tumorgenesis, inflammation, angiogenesis	(193)
Fenretinide	anticancer, chemoprevention, retinal protection	**/**	APCmin/+ mice: 20mg/kg	RAW264.7 HCT116, SW260, SW480, Colo205	↓M2 polarization, Fizz1, PPAR-γ, p-STAT6	inhibit tumorgenesis, angiogenesis	(194)
Plinabulin	anticancer, immune modulation, anti-angiogenesis	**/**	C57BL/6N mice: MC38 subcutaneous inoculation, 7mg/kg	PBMC, BMDM MC38 Hut78	↑ M1 polarization, IL-1β, IL-6, IL12p40, ↓ IL-10, IL-4, ↑ active JNK pathway, Fas-L	inhibit tumor growth, induce tumor cell death through Fas/Fas-L pathway	(195)
Mannose-methyl-β-cyclodextrin	anticancer, immune modulation	**/**	BALB/c male mice: colon-26 subcutaneous inoculation, 10mg/kg	RAW264.7 Colon-26	↓ the amount of M2, ↑ intracellular uptake via MR	inhibit tumor growth, promote tumor cell autophagy	(196)
(Z)-1-(3-((1H-pyrrol-2-yl)methylene)-2-oxoindolin-6-yl)-3-(isoxazol-3-yl)urea derivatives, compound 21	anticancer	**/**	C57BL/6 mice: MC38 subcutaneous inoculation, 5, 10, 20mg/kg	THP-1 MC38, HCT116	↓ M2 polarization, IL-10, ARG1, CHIL3, VEGF, ↑ IL-12, TNF-α, IL-6, ↓ CSF-1R	inhibit tumor growth through suppressing CSF-1R and p-AKT signaling	(197)
Astragaloside IV	immune modulation, anti-inflammatory, antioxidant	aPD-1	BALB/c female mouse: CT26 axillary fat pad injection, Astragaloside IV: 15 mg/kg, aPD-1: 2.5mg/kg	BMDM CT26	↑ M1 polarization, IFN-γ, IL-12, TNF-α, ↓ Arg1, TGF-β, IL-10, VEGF-A	inhibit tumor growth	(184)
the sesquiterpene lactone-rich fraction of I. helenium (SFIH)	anticancer, anti-inflammation, immune modulation	aPD-1	C57BL/6 female mice: MC38 subcutaneous inoculation, SFIH: 50mg/kg, aPD-1: 200μg	**/**	↑ M1 infiltration	inhibit tumor growth, activate adaptive immune response	(198)
Lobeline	anticancer, neuroregulatory effect	aPD-1	C57BL/6 mice: MC38 subcutaneous inoculation, Lobeline: 25mg/kg, aPD1: 5mg/kg	**/**	↑ M1 polarization, ↓ M2 polarization	inhibit tumor growth through regulating MAPK14/p53/Slurp1 signaling pathway, activate adaptive immune response	(149)
Imprime PGG	anticancer, immune modulation	aPD-1	C57BL/6 mice: MC38 subcutaneous inoculation, Imprime: 1.2mg, aPD-1: 0.1mg	**/**	↑ M1 polarization, TNF-α, PD-L1,	inhibit tumor growth, activate NK cells and DC cells, promote T cells expansion	(199)
Chinese yam polysaccharide (CYP)	hypoglycemic, hypolipidemic, immune modulation	aPD-1	C57BL/6 mice: MC38 intraperitoneal injection, BALB/c mice: CT26 intraperitoneal injection, CYP: 100 mg/kg, aPD-1: 200 μg	**/**	↓ M2 polarization, ARG1, IL-10	inhibit tumor growth, regulate intestinal microbiota-related metabolites, reprogram TME	(200)
7S,15R-Dihydroxy-16S,17S-Epoxy-Docosapentaenoic Acid (diHEP-DPA)	anticancer, retinal protection, lipid metabolism regulation	5-FU	BALB/c female mice: CT26 xenograft, diHEP-DPA: 10, 20μg/kg, 5-FU: 20mg/kg	THP-1 HT29, HCT116	↓ M2 infiltration, MMP2, MMP9, VEGF, IL-6, TNF-α, NF-κB signaling pathway, ↑ phagocytic activity via CD47/SIRPα axis	inhibit tumor growth, EMT, inhibit cancer stem cells activation through ROS/STAT3 signaling pathway	(201, 202)
Ovatodiolide	anticancer, anti-inflammation	5-FU	BALB/c mice: CT26 xenograft, Ovatodiolide: 5mg/kg, 5-FU: 30mg/kg	THP-1 HCT116, DLD-1	↓ M2 polarization, ARG1, CD23, YAP1, β-catenin, AKT, NF-κB, IL-6	inhibit tumorigenesis and colon sphere generation	(203)

Aspirin, derived from salicylic acid, is a classic non-steroidal anti-inflammatory drug. Long-term low-dose administration of aspirin (25 mg/kg daily in healthy mice, equivalent to 100 mg in human subjects) significantly reduces macrophage infiltration in colon tumors, shrinks tumor size, and decreases the formation of prostaglandin E2 (PGE2) in the colon (193).Modified mannose β-cyclodextrin (Man-MβCD) can be recognized by mannose receptors (MR) on TAMs, enhancing the cytotoxic activity of M2 macrophages while promoting tumor cell autophagy (196).Plinabulin, a microtubule inhibitor synthesized from a natural product isolated from marine Fusarium fungi through structural optimization, is undergoing global Phase III clinical trials for tumor treatment. *In vivo* studies have shown that Plinabulin (7 mg/kg) induces M1 polarization in macrophages through the JNK signaling pathway, effectively inhibiting tumor growth in MC38 colon cancer-bearing mice (195).The synthetic retinoid derivative fenretinide demonstrates advantages in chemoprevention, significantly reducing tumor occurrence in APCmin/+ mice by inhibiting STAT6 signaling and decreasing M2 macrophage-driven angiogenesis in tumor tissues (194).Blocking CSF-1/CSF-1R signaling to regulate TAM polarization is a promising therapeutic approach. A synthesized compound 21 from a series of (Z)-1-(3-((1H-pyrrol-2-yl)methylene)-2-oxoindolin-6-yl)-3-(isoxazol-3-yl)urea derivatives exhibits excellent CSF-1R inhibitory activity (IC50 = 2.1nM), promoting M1 macrophage infiltration and enhancing anti-tumor immune responses (197).

While synthetic formulations effectively improve the efficacy and stability of natural products, the high research and development costs, along with complex clinical trial processes, remain significant obstacles to their widespread application. Improving the production methods of synthetic formulations, such as utilizing advanced techniques like computer-aided drug design, can aid in the development of synthetic formulations based on natural products.

### 4.3 Combination therapy: immune checkpoint inhibitors and multitarget synergy

Currently, immune checkpoint inhibitors (ICIs, immune checkpoint inhibitors) are working in microsatellite instable/mismatch repair deficient patients that account for about 5-10% of CRC cases. Optimized combination therapy strategies could expand the use of anti-PD-1/PD-L1-based immunotherapy in colon cancer. Although current immunotherapies are mostly centered on T cells, more and more reports support that the effector function of innate immunity is also important for cancer treatment (**Table 4**).

Several studies have explored the synergistic effects of natural products combined with anti-PD-1 (aPD-1) immunotherapy in colon cancer, primarily through the inhibition of TAMs’ M2 polarization and the activation of T-cell immune responses. For example, Astragaloside IV (AS-IV) from Astragalus membranaceus, sesquiterpene lactones from Arctium lappa, and the natural Chinese yam polysaccharide (CYP) have been shown to shift TAMs from the immune-suppressive M2 phenotype to the anti-tumor M1 phenotype. When used in combination with aPD-1, these natural products enhance the tumor immune response, increase the infiltration of CD8+ T cells, and inhibit tumor growth (184, 198, 200). Additionally, CYP has been found to effectively regulate the disruption of gut microbiota and metabolic products associated with aPD-1 treatment (200).

Another promising strategy involves combining natural compounds with conventional chemotherapy agents such as 5-FU. While 5-FU is a cornerstone in the treatment of colon cancer, its efficacy is limited due to the development of chemoresistance. 7S,15R-Dihydroxy-16S,17S-Epoxy-Docosapentaenoic Acid (diHEP-DPA), a DHA derivative, has been shown to overcome 5-FU resistance when used in combination, inhibiting TAM infiltration, reducing the accumulation of cancer stem cells (CSCs), and suppressing EMT (201, 202). Similarly, Ovatodiolide enhances anti-tumor effects when combined with 5-FU by inhibiting the YAP1 oncogenic pathway and preventing M2 TAM polarization (203).

The combination of natural products with existing cancer therapies (such as ICIs and chemotherapy) offers multiple advantages, particularly in enhancing current therapeutic efficacy, overcoming resistance, and reducing side effects. However, current research has primarily focused on the combination of natural products with anti-PD-1 immunotherapy, with limited studies on the use of other immunotherapies in combination with natural products for colon cancer treatment. Future research should explore the synergistic effects of different immune checkpoint inhibitors with natural products to provide more treatment options for clinical practice.

### 4.4 Nanomedicine strategies involving natural products

Nanomedicines have shown great potential in cancer treatment and have become a promising strategy to overcome the pharmacokinetic limitations of natural compounds. Despite substantial evidence supporting the superior antitumor effects of natural products, their clinical application remains limited due to issues such as poor solubility, low bioavailability, and insufficient systemic stability. The rapid development of functional nanomaterials has enabled nanomedicines to effectively address these challenges by facilitating accurate drug release, enhancing tumor penetration, and improving retention through the enhanced permeability and retention (EPR) effect, all while reducing toxicity and side effects (204). Common carriers include biomimetic delivery platforms (e.g., erythrocyte membranes, leukocyte membranes, stem cell membranes, and extracellular vesicles), liposomes, polymeric particles, and inorganic nanoparticles. Preclinical studies suggest that TAM-targeting nanomedicines exhibit good safety, enhanced stability, and improved anticancer performance in colon cancer treatment (**Table 5**).

**Table 5:** Natural product–based nanomedicines targeting TAMs for colon cancer therapy.

Type	Biofilm/host material	Drugs	Experimental model	Effect on the macrophages	Function	Ref.
RSV-NPs@RBCm	erythrocyte membrane	Resveratrol	*in vitro* and *in vivo*	↑ escape macrophage phagocytosis	suppress tumor growth via promoting tumor cells ferroptosis	(205)
HPA/AS/CQ@Man-EM	erythrocyte membrane/PLGA	Artesunate and Chloroquine	*in vitro* and *in vivo*	↑ M1 polarization, TNF-α, IFN-γ, IL-6, IRF5, LC3II/LC3I, NF-κB, p-p38↓ M2 polarization, TGF-β, ARG1, IL-10	suppress tumor growth and angiogenesis via upregulating ROS level and downregulating VEGF protein level	(206)
GDNPs	extracellular vesicle-liked nanoparticles	Ginseng	*in vitro* and *in vivo*	↓ M2 polarization, ARG1	suppress tumor growth, promote proliferation and activation of T cells through mTOR-bet axis	(207)
M1EVs	macrophage 1-derived extracellular vesicles	Oxaliplatin, Retinoic acid, and Libidibia ferrea	*in vitro* and *in vivo*	↓ M2 polarization, IL-10, TGF-β, CCL22, STAT3/NF-κB/AKT pathway	suppress tumor growth, EMT, metastasis, promote CD8+ T cells infiltration	(208)
CaZCH NPs	hyaluronic acid- modified	Ca2+, Curcumin and H2O2	*in vitro* and *in vivo*	↑ M1 polarization	suppress tumor growth, induce tumor cells immunogenic ferroptosis, alleviate the immunosuppressive TME	(209)
MPLO@HA	hyaluronic acid- modified	Metformin, Oxaliplatin and Lauric acid	*in vitro* and *in vivo*	↑ M1 polarization, IL-12, ↓ IL-10	suppress tumor growth, induce tumor cells ICD, inhibit Tregs and MDSCs	(210)
M2 peptide-modified nanoliposomes containing crocin	liposome	Crocin	*in vitro* and *in vivo*	↑ M1 polarization, TNF-α, IL-12β	suppress tumor growth	(211)
LNPs	liposome	Mulberry Leaf	*in vitro* and *in vivo*	↑ M2 polarization, IL-10, ↓ TNF-α, IL-6, CD98, ↑ Galectin-3, PI3K/STAT6 pathway	suppress tumor progression, alleviate inflammation	(212)
TS-NP	egg phosphatidylcholine	Tocopheryl succinate	*in vivo*	↓ M2 polarization, VEGF-A, IL-10	suppress tumor growth and metastasis	(213)
Apt-2cNP	PLGA	Betulinic acid	*in vitro* and *in vivo*	↑ M1 polarization	suppress tumor growth, induce tumor cells apoptosis and autophagy, promote immune cells activation	(214)
CHO-PLGA-RA NPs	PLGA NPs coated with cholesterol	Retinoic acid	*in vitro* and *in vivo*	↓ M2 polarization, NF-κB, STAT3, IL-10, TGF-β	suppress tumor growth, EMT, metastasis, promote CD8+ T cells infiltration	(215)
EBNPs	poly (ethylene oxide)-poly (butylene oxide)	Linoleic acid conjugated SN38	*in vitro*	↑ escape macrophage phagocytosis	suppress tumor growth, promote tumor cells apoptosis	(216)
SeNps	selenium nanoparticles	Lactobacillus casei ATCC 393	*in vitro*	↑ macrophage phagocytosis of tumor cells	promote tumor cells apoptosis and ICD	(217)
LNT-UA	/	Ursolic acid and Lentinan	*in vitro* and *in vivo*	↑ M1 polarization, IFN-γ, TNF-α, ↓ IL-10	suppress tumor growth and metastasis, induce tumor cells ICD, promote activation of DC and recruitment of T cells	(218)

#### 4.4.1 Biomimetic delivery systems

Cell membrane-coated nanoparticles, which mimic the structure of cell membranes, can enhance biocompatibility and improve drug targeting. For example, erythrocyte membrane-coated nanoparticles (RSV-NPs@RBCm) effectively evade macrophage phagocytosis, extend the circulation time of drugs in the bloodstream, and improve tumor-targeted delivery through tumor-penetrating peptides (205). Extracellular vesicle-like nanoparticles derived from fresh ginseng can inhibit M2 polarization, modulating arginine metabolism to affect the mTOR-T-bet axis, thereby effectively alleviating T cell exhaustion (207). Furthermore, macrophage-derived extracellular vesicle nanoparticles possess excellent biocompatibility, enabling efficient drug loading and inhibition of colorectal cancer cell proliferation, migration, and resistance (208).

#### 4.4.2 Liposomes

Liposomes, due to their excellent biocompatibility and biodegradability, are widely used as classic nanocarriers. Their structure consists of an outer lipid bilayer and an internal hydrophilic core. For example, saffron extract-loaded liposomes modified with M2 peptides specifically target M2 macrophages in the colorectal TME, inducing their polarization to the M1 phenotype, thereby exerting antitumor effects (211). Additionally, tocopheryl succinate succinate nanoparticles synthesized with egg phosphatidylcholine prevent peritoneal dissemination caused by colon cancer through intraperitoneal injection. This mechanism may involve the suppression of M2 macrophages, reducing the release of IL-10 and VEGF, and improving the TME (213).

#### 4.4.3 Inorganic nanomaterials

Inorganic nanomaterials, such as metal nanoparticles, metal oxides, and carbon-based nanomaterials, are widely utilized in tumor therapy due to their biological stability and targeting capabilities. For instance, CaZCH NPs, which respond to the acidic conditions of the tumor microenvironment, release active substances that promote the infiltration of M1 macrophages and enhance antitumor immune responses by inducing ICD (209).

#### 4.4.4 Polymeric nanomaterials

Poly(lactic-co-glycolic acid) (PLGA) is a biodegradable and controlled-release drug delivery system approved by the FDA for sustained drug formulations. Natural product-based formulations, such as Apt-2cNP and CHO-PLGA-RA NPs, utilize PLGA as a carrier to modulate TAM polarization within the colorectal TME, inhibiting the NF-κB/STAT3 signaling pathway associated with M2 polarization and reshaping the immunosuppressive microenvironment (214, 215). Moreover, EBNPs avoid macrophage phagocytosis, effectively promoting cancer cell uptake, and achieving targeted inhibition of colon cancer cells (216).

Studies show that macrophage-targeting nanomedicines based on natural products have significant advantages. However, challenges remain, including instability in distribution, inter-individual variations in the EPR effect, and off-target effects. Furthermore, the heterogeneity of TAMs (including different subtypes, such as resident *vs*. recruited, anti-inflammatory *vs*. pro-inflammatory) influences therapeutic outcomes. The pharmacokinetic interactions between nanomedicines, tumor cells, and macrophages require further investigation. Additionally, animal models cannot fully replicate the complexity of the human tumor microenvironment. Future research should focus on improving the selective delivery of nanoparticles, reducing off-target toxicity, and enhancing therapeutic outcomes through more accurate pharmacokinetic modeling. Furthermore, in-depth studies on TAM heterogeneity and strategies for targeting different TAM subtypes will contribute to advancing the clinical application of nanomedicines.

### 4.5 Traditional Chinese medicine formulas: multitarget synergistic antitumor effects

Traditional Chinese medicines effectively inhibit the development of colon cancer through various pathways, such as enhancing immune response and inhibiting tumor proliferation and metastasis. Garcinia yunnanensis, a traditional Chinese medicine, has anticancer and anti-inflammatory effects, and after Garcinia yunnanensis treatment of APCmin/+ colon cancer model mice, JNK, STAT3 and ERK signaling were inhibited, M2 macrophage infiltration in the TME was reduced, and the size of tumors was reduced (219). Yi-Yi-Fu-Zi-Bai-Jiang-San (YYFZBJS) is a commonly used traditional Chinese medicine formula for treating inflammatory diseases. YYFZBJS can alter the pro-tumor effects of M2 macrophages by inhibiting inflammation-related signaling pathways in colon cancer and delay the development of the disease by reshaping the gut microbiota (220). Si-Jun-Zi Decoction (SJZ) is a well-known Chinese herbal formula that can regulate human immunity. After 3 weeks of treatment with full dose (45 g/kg) of modified SJZ in mice with colorectal cancer, the expression level of GM-CSF in plasma and the number of splenic macrophages were significantly increased, and the nude mice had better body defenses and colorectal cancer survival rate, and the modified SJZ also reduced the hepatic metastasis of colorectal cancer by activating the innate immunity (221).

The development of malignant tumors can have a deleterious effect on the patient’s mood and even accelerate tumor metastasis, and some traditional Chinese medicines can slow down this process. Xiao-Yao-San, is widely used clinically for the relief of symptoms associated with depression. Treatment of colorectal model mice with Xiao-Yao-San reduced the recruitment of M2 macrophages and MDSCs, and inhibited the expression of TGF-β, IL-6, MMP-9 and VEGF. Not only that, Xiao-Yao-San can also effectively inhibit liver metastasis of chronic stress colon tumors (222). Hereby, the natural products, with their excellent activity, safety and novel structures, offer more possibilities for the development of novel anti-colon cancer drugs.

### 4.6 Probiotics and gut microbiome regulation

Probiotics are defined by the World Health Organization (WHO) as “active microorganisms that, when ingested in sufficient quantities, are beneficial to the health of the host”. Probiotics stabilize the intestinal barrier and modulate intestinal immunity. Extensive research has shown that the consumption of probiotics (e.g. Lactobacillus plantarum, Lactobacillus acidophilus, and Bacillus subtilis) can alter the distribution of cellular junctional proteins, reduce the absorption of potentially cancer-causing compounds, and improve the poor prognosis of colon cancer (223–226).

lactobacilli, a probiotic used with high frequency in the treatment of diseases. Hradicka et al. found that Lactobacillus has immunomodulatory activity, remodels macrophage polarization, and reduces colon tumor tissue volume and total tumor number (227). Lactobacillus acidophilus lysate in combination with CTLA-4 helps to enhance anti-tumor immune response in colon cancer mice by decreasing Treg and M2 macrophage infiltration, and increasing the number of CD8+ T cells in the TME (228). Saccharomyces cerevisiae (S. cerevisiae) is a conventional yeast used in food products and a probiotic that maintains intestinal homeostasis. Beta-glucan, a major component of the cell wall of S. cerevisiae, inhibits colon tumor activity by suppressing intestinal ecological dysregulation and activating macrophages (229). Loigolactobacillus coryniformis NA-3 induces NO, IL-6, TNF-α, and ROS production in colonic TAMs and significantly inhibits the proliferation of colonic tumor cells (230). Notably, appropriate dosage and sufficient intestinal residence time are crucial for probiotics to exert anticancer immune effects (231). In addition, several studies have confirmed that the use of probiotics can help alleviate gastrointestinal adverse reactions such as diarrhea and nausea caused by chemotherapeutic drugs and improve patients’ treatment tolerance (231–233).

### 4.7 Dietary intervention

Dietary intervention is an emerging strategy for disease prevention and treatment. A Western diet characterized by high intake of red meat and high fat has been shown to significantly increase the risk of colorectal cancer (234). In contrast, diets rich in dietary fiber have a protective effect on the gut, not only providing an energy source for gut microbes, but also helping to maintain gut homeostasis and barrier integrity. Compared to normal-diet colon cancer model mice, ketogenic diet induces oxidative stress within colon tumors, promotes conversion of M2 macrophages to M1-type, and downregulates MMP expression to inhibit colon tumor progression (235). In addition, some dietary intake can also alleviate colon cancer progression to some extent. Feeding a diet rich in Oleuropein-Rich Leaf Extract to colon cancer model rats abrogated the overexpression of iNOS in rat tumors and inhibited the pro-inflammatory behaviors of macrophages in rats with loaded tumors, suppressing tumor proliferation (190). 3-Hydroxybutyrate (3HB) has various utilities as an oral food supplement, such as energy supply, anti-inflammation, neuroprotection, and can be used to improve alcoholic fatty liver, osteoporosis, and Alzheimer’s disease. Li et al. found that 3HB could regulate colon TME, modulate the proportion of infiltrating macrophages, and reduce the level of MDSCs, as well as inhibit the NF-κB pathway that reduces inflammatory factor release (236). Sea cucumber, a marine animal rich in bioactive molecules, is a dietary nutrient with anti-inflammatory and antibacterial effects. Adding sea cucumber extract Frondanol A5 to the feed fed to APCmin/+mice significantly reduced the volume of colon tumors, increased the phagocytosis efficiency of mouse peritoneal macrophages, and provided prevention for intestinal tumorigenesis (237).

## 5 Challenges and future perspectives

**Figure 4:**
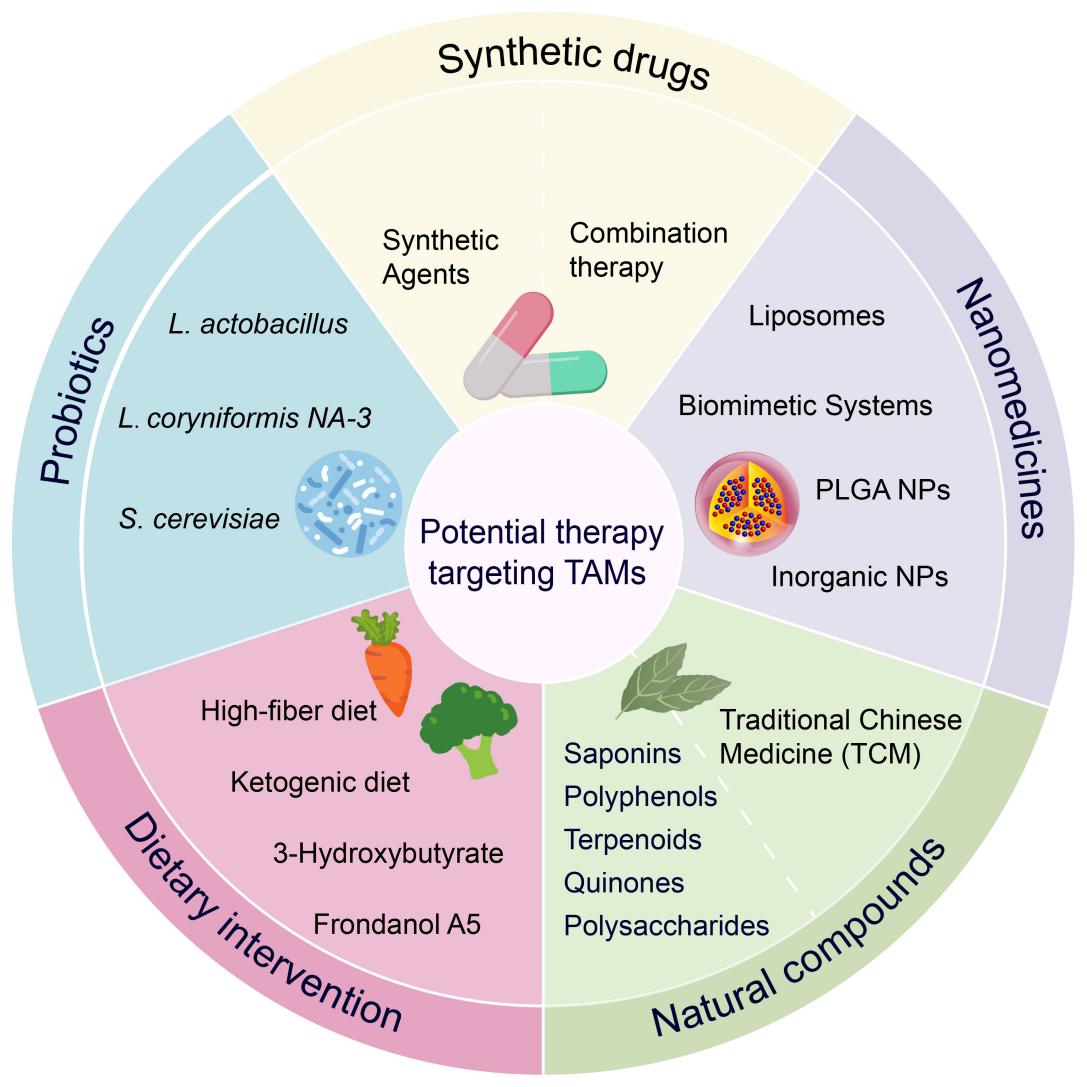
Potential strategies for targeting TAMs in the treatment of colon cancer.

In this review, we summarize the potential of targeted modulation of TAMs in colon cancer prevention, occurrence, metastasis and drug resistance. The main pathways include modulation of TAMs infiltration in TME, remodeling of TAMs polarization, modulation of key signaling pathways in TAMs, promotion of phagocytic activity of TAMs, and activation of immunomodulatory function of TAMs. Potential therapeutic strategies include drug treatments based on natural products and dietary interventions (**Figure 4**). Colon carcinogenesis is a heterogeneous process with a diverse array of somatic molecular alterations influenced by diet, environmental and microbial exposures, and host immunity. With the deepening of research on colon cancer and the emergence of new therapeutic modalities, especially targeted therapies and immunotherapies, coupled with increasing early interventions, colon cancer has made great progress in diagnosis and treatment. However, toxic side effects, drug resistance, off-target effects, high metastasis and recurrence rates resulting from treatment are still important factors threatening human health.

Based on the current challenges in colon cancer treatment, it is particularly important to improve efficacy and overcome drug resistance without exacerbating immune-related side effects. A growing body of findings suggests that targeting macrophages for colon cancer treatment is a promising therapeutic approach.

However, there are some unresolved issues in the current studies on macrophages in colon cancer, and despite the large number of studies supporting that macrophage infiltration is suggestive of a poor prognosis, there are still data supporting the opposite result, which may be related to the lack of clarity of macrophage markers as well as difficulty in identifying the phenotype. Due to the tumor heterogeneity and plasticity of macrophages, the mechanism of how to inhibit macrophages from being “educated” by TME to perform their scavenging role to phagocytose tumor cells needs to be explored in greater depth. Macrophages signaling pathways and key regulatory factors affecting macrophages in colon cancer also need to be further revealed.

We have summarized potential natural drugs for targeting TAMs in the treatment of colon cancer. These include botanical active compounds, natural product-based synthetic formulations, therapies combined with existing anticancer treatments (such as ICIs and chemotherapy), natural product-based nanomedicines, probiotics, traditional Chinese medicine, and dietary interventions. These therapeutic strategies have addressed issues such as single efficacy, off-target effects, and drug resistance to some extent. Compared with traditional chemotherapy drugs, natural products tend to have fewer adverse effects and toxicities. Additionally, some traditional medicines can inhibit the growth of colon tumors by improving the body’s stress response. However, there are several challenges regarding the clinical application of natural products. Many phytochemicals exhibit poor solubility, resulting in low bioavailability. The bioavailability of some natural compounds remains unclear, and their metabolic pathways and transformation processes *in vivo* are not fully understood. Although progress has been made in improving the drug delivery efficiency and bioavailability of natural product-based nanomedicines and synthetic formulations, most studies are still in the basic experimental stage, and sufficient clinical data to support widespread application is lacking.

Natural products targeting TAMs for colon cancer treatment show great potential. Future research should focus on addressing key issues such as solubility, delivery efficiency, and bioavailability of natural products, and conduct more clinical trials to verify their efficacy and safety. As cancer biology continues to advance, these challenges are expected to be overcome through further technological innovations and research, ultimately contributing to cancer treatment and human health.
